# Opposite Regulation of CHOP and GRP78 and Synergistic Apoptosis Induction by Selenium Yeast and Fish Oil via AMPK Activation in Lung Adenocarcinoma Cells

**DOI:** 10.3390/nu10101458

**Published:** 2018-10-08

**Authors:** Ruey-Ho Kao, Gi-Ming Lai, Jyh-Ming Chow, Chien-Huang Liao, Yu-Mei Zheng, Wei-Lun Tsai, Simon Hsia, I-Chun Lai, Hsin-Lun Lee, Shuang-En Chuang, Jacqueline Whang-Peng, Chih-Jung Yao

**Affiliations:** 1 Cancer Center, Wan Fang Hospital, Taipei Medical University, Taipei 11696, Taiwan; rueykao@gmail.com (R.-H.K.); gminlai@nhri.org.tw (G.-M.L.); chow0803@yahoo.com.tw (J.-M.C.); a2639264@ms25.hinet.net (C.-H.L.); clairgg@hotmail.com (W.-L.T.); jqwpeng@nhri.org.tw (J.W.-P.); 2 Division of Hematology and Medical Oncology, Department of Internal Medicine, Wan Fang Hospital, Taipei Medical University, Taipei 11696, Taiwan; lilindr3@gmail.com; 3 Taipei Cancer Center, Taipei Medical University, Taipei 11031, Taiwan; 4 Department of Internal Medicine, School of Medicine, College of Medicine, Taipei Medical University, Taipei 11031, Taiwan; 5 National Institute of Cancer Research, National Health Research Institutes, Miaoli 35053, Taiwan; sechuang@nhri.org.tw; 6 Taiwan Nutraceutical Association, Taipei 10596, Taiwan; Dr.Simon.hsia@gmail.com; 7 Division of Radiation Oncology, Department of Oncology, Taipei Veterans General Hospital, Taipei 11217, Taiwan; littlelai0114@gmail.com; 8 Department of Radiation Oncology, Taipei Medical University Hospital, Taipei Medical University, Taipei 11031, Taiwan; b001089024@tmu.edu.tw

**Keywords:** selenium, fish oil, lung adenocarcinoma, ER stress, apoptosis

## Abstract

Selenium has been intensively studied for the use of cancer prevention and treatment. However, the clinical effects are still plausible. To enhance its efficacy, a combinational study of selenium yeast (SY) and fish oil (FO) was performed in A549, CL1-0, H1299, HCC827 lung adenocarcinoma (LADC) cells to investigate the enhancement in apoptosis induction and underlying mechanism. By sulforhodamine B staining, Western blot and flow cytometric assays, we found a synergism between SY and FO in growth inhibition and apoptosis induction of LADC cells. In contrast, the fetal lung fibroblast cells (MRC-5) were unsusceptible to this combination effect. FO synergized SY-induced apoptosis of A549 cells, accompanied with synergistic activation of AMP-activated protein kinase (AMPK) and reduction of Cyclooxygenase (COX)-2 and β-catenin. Particularly, combining with FO not only enhanced the SY-elevated proapoptotic endoplasmic reticulum (ER) stress marker CCAAT/enhancer-binding protein homologous protein (CHOP), but also reduced the cytoprotective glucose regulated protein of molecular weight 78 kDa (GRP78). Consequently, the CHOP downstream targets such as phospho-JNK and death receptor 5 were also elevated, along with the cleavage of caspase-8, -3, and the ER stress-related caspase-4. Accordingly, inhibition of AMPK by compound C diminished the synergistic apoptosis induction, and elevated CHOP/GRP78 ratio by SY combined with FO. The AMPK-dependent synergism suggests the combination of SY and FO for chemoprevention and integrative treatment of LADC.

## 1. Introduction

Lung cancer is the leading cause of cancer-related mortality worldwide, and its rate of incidence continues increasing [1]. Lung adenocarcinoma (LADC) is the most common type of lung cancer, and the five years survival rate is still dismal, in spite of recent advances in targeted therapy for its treatment [2]. New approaches to tackle this disease are urgently needed. In addition to the synthesis of new cytotoxic compounds or tyrosine kinase inhibitors, the evidence-based anticancer nutraceuticals, with a wide safety margin, represents a potential resource of candidates for this approach.

The trace element selenium is an essential nutraceutical for human health and has attracted a great attention, because of its potential in cancer treatment and chemoprevention [3,4]. Selenium compounds have been intensively studied, in many experimental models, against several malignancies, including lung cancer [3]. A variety of clinical trials were conducted to evaluate the safety and efficacy of selenium in lung cancers patients [5,6]. Although a recent study of meta-analysis and meta-regression concluded that high selenium exposure decreased the risk of lung cancer, as well as other four types of cancers (breast, esophageal, gastric and prostate) [6], some outcomes of other trials were not as favorable as expected or even conflict [5,7]. As such, selenium supplementation was not recommended as a general strategy for lung cancer prevention [5]. As the potency of a single dietary component might be limited, further combinational study was thus prompted to enhance the efficacy of selenium for the prevention and treatment of lung cancer.

Various mechanisms, such as apoptosis induction, proliferation inhibition, redox state modulation, carcinogen detoxification, immune system stimulation and angiogenesis suppression have been proposed to explain the anticancer activities of selenium. Among those mechanisms, induction of apoptosis has been regarded as the critical cellular event in cancer chemoprevention and chemotherapy by selenium compounds [8,9]. Numerous studies have demonstrated that AMP-activated protein kinase (AMPK) activation plays a key role in selenium-induced apoptosis of cancer cells [10,11,12], which in turn suppresses oncoproteins such as Cyclooxygenase (COX-2) [11] and β-catenin [10]. Further mechanistic studies delineated that cancer cell apoptosis induced by selenium is linked to the endoplasmic reticulum (ER) stress/unfolded protein response (UPR)-apoptosis cascade [13,14,15]. Selenium elicits the apoptotic ER stress marker, such as CHOP (CAAT/enhancer binding protein homologous protein or growth arrest DNA damage inducible gene *153*, *GADD153*), along with the activation of human caspase-4 (rodent caspase-12 homolog) [13,15], which has been specifically involved in ER stress-induced apoptosis [13,14,15]. In general, a higher concentration of selenium was required to elicit the apoptotic ER stress marker like CHOP compared with the survival marker, such as glucose regulated protein of molecular weight 78 kDa (GRP78) [15]. When the dose of selenium is increased to the effective level, the rescue effort of cancer cells will fail to cope with and recover from ER stress, resulting in the commitment to apoptosis [15]. Therefore, combination with another nutraceutical to enhance the activation of AMPK and raise the ratio of CHOP to GRP78 during supplementation challenge with selenium may practically improve its efficacy in cancer prevention and treatment.

Fish oil (FO) is a dietary supplement containing omega-3 fatty acids, particularly docosahexaenoic acid (DHA) and eicosapentaenoic acid (EPA), which suppress COX-2 [16], and has beneficial cardiovascular effects [17]. Given that a growing body of evidence indicates the etiological impact of COX-2 in cancer, FO, as well as DHA and EPA, also have been reported to exert anti-neoplastic activities, playing a potential role either in cancer prevention or in cancer therapy [16]. Several basic studies have demonstrated the effects of FO omega-3 fatty acids against lung cancer [16,18,19] and clinical studies have shown their effects to improve the outcomes of chemotherapy in lung cancer patients [20]. In addition to invasion and metastasis inhibition, apoptosis induction also has been suggested to underlie the anticancer effects of FO omega-3 fatty acids [16]. Moreover, like selenium, their apoptosis induction effects in cancer cells are also involved with the activation of AMPK [16,18,21,22] and caspase-4 [23], as well as the modulation of ER stress markers, such as CHOP [23] and GRP78 [24].

Therefore, it is interesting to investigate the combination effects of selenium and FO omega-3 fatty acids on the apoptosis induction in lung cancer cells. The selenium compounds used in preclinical and clinical studies can be broadly categorized into organic and inorganic forms. Animal data have shown that the organic forms of selenium are both safer and more effective than the commonly-used inorganic forms, such as sodium selenite [3]. A recognized source of organic food-form is the selenium yeast (SY), which is produced by growing select strains of *Saccharomyces cerevisiae* in selenium-rich media [25]. It predominantly contains l-selenomethionine [3] and the safety record is excellent [25]. In the present study, we found a synergistic apoptotic response between SY and FO in human LADC cell lines. To elucidate the underlying mechanism, the combination effects of SY and FO on the activation of AMPK, and modulation of ER stress markers (CHOP and GRP78), and downstream targets in LADC cells were investigated.

## 2. Materials and Methods

### 2.1. Cell Culture

The human LADC cell lines A549, CL1-0 and HCC827 were maintained in Roswell Park Memorial Institute (RPMI)-1640 (Gibco, Carlsbad, CA, USA), and H1299 was in Dulbecco’s Modified Eagle’s medium (DMEM) (Gibco, Carlsbad, CA, USA). The human fetal lung fibroblast MRC-5 cells were cultured in Eagle’s Minimum Essential Medium (EMEM) (Gibco, Carlsbad, CA, USA). All the mediums were supplemented with 10% fetal bovine serum (Corning Incorporated, Corning, NY, USA), 1× penicillin-streptomycin-glutamine (Corning Incorporated, Corning, NY, USA), and 1× nonessential amino acids (Corning Incorporated, Corning, NY, USA). Cells were cultured at 37 °C, in a water-jacketed 5% CO_2_ incubator. The A549, HCC827 and H1299 cells were purchased from the American Type Culture Collection (Manassas, VA, USA) and the CL1-0 cells were kindly provided by Dr. Shine-Gwo Shiah (National Health Research Institutes, Miaoli, Taiwan). The MRC-5 normal fetal human lung fibroblasts were purchased from Bioresource Collection and Research Center (Hsinchu, Taiwan).

### 2.2. Reagents and Chemicals

The stock solutions of Selenium yeast (SY) and fish oil (FO) (each gram contains 220 mg DHA and 330 mg EPA) were provided by Dr. Chih-Hung Guo (Institute of Biomedical Nutrition, Hung-Kuang University, Taichung, Taiwan). They were then aliquoted and stored at −20 °C (FO) and −80 °C (SY), respectively. Both of them were diluted in sterile culture medium immediately prior to use. The concentration of FO mentioned in the text represents its omega-3 fatty acid (DHA + EPA) content. Sulforhodamine B (SRB), trichloroacetic acid, propidium iodide and compound C (also called dorsomorphin, an AMPK inhibitor) were purchased from Sigma-Aldrich, Co. (St. Louis, MO, USA). FITC Annexin V Apoptosis Detection Kit with 7-amino-actinomycin D (7-AAD) was from BioLegend Inc. (San Diego, CA, USA).

### 2.3. Measurement of Cell Viability

The cells were seeded in 96-well plates for 24 h (A549, 1500 cells/well; CL1-0, H1299 and HCC827, 2000 cells/well; MRC-5, 2500 cells/well) and then treated with drugs or sterile culture medium for 72 h. The cell viability was measured with SRB binding assay. Briefly, the cells were fixed with 10% trichloroacetic acid and incubated for 1 h, at 4 °C. The plates were then washed twice with tap water and air dried. The dried plates were stained with 80 µL of 0.4% (*w*/*v*) SRB prepared in 1% (*v*/*v*) acetic acid for 30 min at room temperature. The plates were rinsed quickly, twice with 1% acetic acid to remove unbound SRB, and then air dried until no moisture was visible. The bound dye was solubilized in 20 mmol/L Tris base (200 µL/well) for 5 min on a shaker. Optical densities were read on a microplate reader ELx800 (BioTek Instruments, Inc., Winooski, VT, USA) at 570 nm. The optical density is directly proportional to the cell number over a wide range.

### 2.4. Photograph of the Cells

The phase contrast and bright field for trypan blue exclusion assay images of cells were photographed using a digital microscope camera PAXcam2+ (Midwest Information Systems, Inc., Villa Park, IL, USA) adapted to an inverted microscope CKX31 (Olympus Co., Tokyo, Japan) at 20× objective lens magnification.

### 2.5. Analysis of Apoptotic and Necrotic Cell Death by Annexin V/7-Amino-Actinomycin D Staining

One day after being seeded in 6 cm dish (8 × 10^4^ cells/dish), the A549 cells were treated with agents as indicated in the figure for 72 h. The treated cells were analyzed by annexin V/7-amino-actinomycin D (7-AAD) staining to examine the induction of apoptosis and necrosis. Annexin V/7-AAD staining is a prevalent method for discriminating early apoptosis from late apoptosis and necrosis. Early apoptotic cells still maintain the plasma membrane integrity, which excludes the vital dyes, such as 7-AAD (DNA intercalator), but the phosphatidylserines (PS) on the outer leaflet of the plasma membrane can be stained by fluorescein-labelled annexin V. In contrast, late apoptotic cells and necrotic cells lose their cell membrane integrity and can be stained by 7-AAD [26]. After treatment, cells were washed twice with cold Cell Staining Buffer (BioLegend, Inc., San Diego, CA, USA), and then resuspended cells in Annexin V Binding Buffer (BioLegend, Inc., San Diego, CA, USA) at a concentration of 1.6 × 10^6^ cells/mL. Transfer 100 µL of the cell suspension to a 5 mL test tube and then add 5 µL of FITC Annexin V (BioLegend, Inc., San Diego, CA, USA) and 5 µL of 7-AAD Viability Staining Solution. After being gently vortexed and incubated for 15 min at room temperature (25 °C) in the dark, 400 µL of Annexin V Binding Buffer (BioLegend, Inc., San Diego, CA, USA) was add to the tube and then the cells were analyzed by CytoFLEX flow cytometer (Beckman Coulter, Inc., Indianapolis, IN, USA). A minimum of 10,000 events were collected and analyzed.

### 2.6. Analysis of Apoptotic Sub-G1 Fraction by Propidium Iodide Staining

One day after being seeded in 6-cm dish (A549 cells, 8 × 10^4^ cells/dish; CL1-0, H1299 and HCC827cells, 9 × 10^4^ cells/dish), the cells were treated with agents as indicated in the figure for 72 h. At harvest, cells were fixed in ice-cold 70% ethanol and stored at −20 °C. Cells were then washed twice with ice-cold phosphate-buffered saline and then incubated with RNase and DNA intercalating dye propidium iodide (50 µg/mL) at room temperature for 20 min. The percentages of apoptotic sub-G1 fraction were then analyzed using a Cytoflex flow cytometer (Beckman Coulter, Inc., Indianapolis, IN, USA). A minimum of 10,000 events were collected and analyzed.

### 2.7. Western Blot

After being seeded in 10 cm dishes at a density of 3.5 × 10^5^ cells/dish for 24 h, A549 cells were then treated with agents as described in the figures. On the day of harvest, the whole-cell lysates were extracted with 1× radioimmunoprecipitation lysis buffer (Merck Millipore, Billerica, MA, USA) containing 1× tyrosine phosphatase inhibitor cocktail FC0020-0001(BIONOVAS, Toronto, ON, Canada), 1× protease inhibitor cocktail, full range (FC0070-0001, BIONOVAS, Toronto, ON, Canada), and 1× serine/threonine phosphatase inhibitor cocktail (FC0030-0001, BIONOVAS, Toronto, ON, Canada). The protein extracts were resolved by sodium dodecyl sulfate–polyacrylamide gel electrophoresis and subsequently transferred to polyvinylidene difluoride membrane (GE Healthcare, Pittsburgh, PA, USA) by electroblotting. The membranes were blocked with 5% bovine serum albumin in Tris-buffered saline (TBST) buffer (Tris-buffered saline with Tween 20, 25 mM Tris-HCl, 125 mM NaCl, 0.1% Tween 20) for 1 h at room temperature and incubated with primary antibody overnight at 4 °C and then with horseradish peroxidase-conjugated secondary antibody for 1 h at room temperature. Intensive wash with TBST buffer was performed after each time of incubation. The immune complexes were visualized using enhanced Chemiluminescence (ECL) Reagent Plus (Perkin Elmer, Inc., Waltham, MA, USA) on the Syngene G:Box chemi XL gel documentation system (Syngene, Cambridge, UK) according to the manufacturer’s instructions. Quantification of Western blot band intensities was performed using ImageJ software (ImageJ bundled with 64-bit Java 1.8.0_112, National Institutes of Health, Bethesda, MD, USA) downloaded from https://imagej.nih.gov/ij/download.html.

### 2.8. Antibodies

Primary antibodies against phospho-AMPKα (Thr172, #2535), non-phospho (Active) β-Catenin (Ser33/37/Thr41, #8814), CHOP (#2895), death receptor-5 (DR5) (#3696), phospho-JNK (#9255), cleaved caspase-9 (Asp315, #9505), and cleaved caspase-3 (Asp175, #9664) were purchased from Cell Signaling Technology, Inc. (Danvers, MA, USA). Primary antibodies for cleaved caspase-4 (ab75182), COX-2 (Ab62331) and Glyceraldehyde-3-Phosphate Dehydrogenase (GAPDH) (Ab8245) were purchased from Abcam, Inc. (Cambridge, MA, USA). Primary antibody for GRP78 (#PA1-014A) was purchased from Thermo Fisher Scientific™ (Waltham, MA, USA). Primary antibody for full-length caspase-8 (GTX110723) was from GeneTex, Inc. (Irvine, CA, USA) phospho-IRE1 (#3881) was from Epitomics, Inc. (Burlingame, CA, USA) and phospho-PERK (Thr980, bs-3330R) was from Bioss Antibodies (Woburn, MA, USA), respectively.

### 2.9. Analysis of Synergistic Combination Effect

The synergism between SY and FO on the growth inhibition of cancer cells was analyzed by the combination-index (CI) derived from the median effect principle of Chou and Talalay [27], using the CalcuSyn software (version 1.1.1; Biosoft, Cambridge, UK). The value of CI = 1 indicates an additive effect, whereas the value of CI < 1 or CI > 1 indicates, synergism or antagonism, respectively.

### 2.10. Statistical Analysis

The quantitative Western blot data were expressed as the mean ± standard error of the mean (SEM), and analyzed using unpaired Student’s *t*-test for comparison between two groups. Differences with a *p* value < 0.05 were considered statistically significant.

## 3. Results

### 3.1. SY and FO Act Synergistically to Inhibit the Growth of LADC Cells

**Figure 1:**
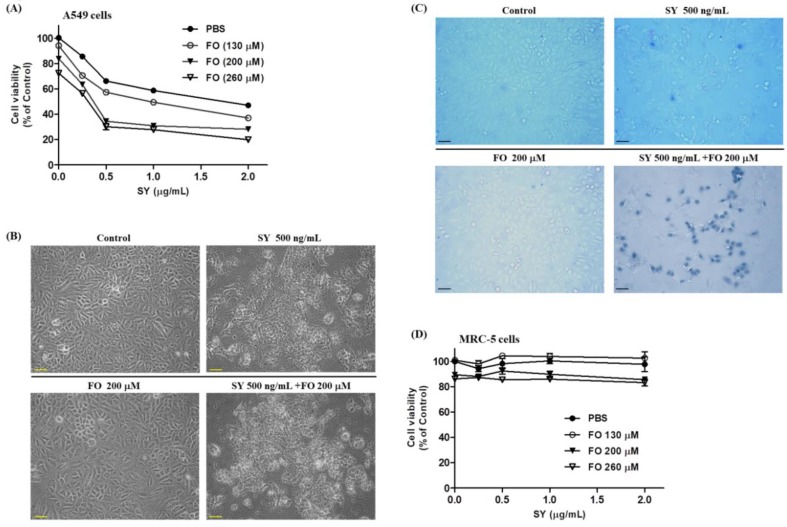
Combination effects of selenium yeast (SY) and fish oil (FO) on the cell viabilities of human lung adenocarcinoma A549 and human fetal lung fibroblast (MRC)-5 cells. Cells were treated with the indicated concentrations of SY and FO for 72 h as described in materials and methods (Section 2). (**A**) Cell viabilities of A549 cells determined by sulforhodamine B binding assay. Data are expressed as mean ± standard error. (**B**) Representative phase-contrast microscopy images of the A549 cells, scale bar = 50 µm. (**C**) Representative bright-field microscopy images of the A549 cells stained by trypan blue (0.2%), scale bar = 50 µm. (**D**) Cell viabilities of MRC-5 cells determined by sulforhodamine B binding assay. PBS: phosphate buffered saline.

The effects of SY and FO, either alone or in combination, on the proliferation of LADC cells were determined by the SRB assay (Figure 1). After 72 h of treatment, SY only slightly reduced the proliferation of A549 cells to 66.2% of control at concentration of 500 ng/mL and dose-dependently decreased the proliferation to 47% at concentration of 2000 ng/mL (Figure 1A). However, it had been reported that 500 ng/mL was the upper limit of the mean plasma selenium levels associated with toxicity in human [28]. To enhance the effect of SY at this safe concentration, we examined its combination effect with FO. As shown in Figure 1A, the proliferation of 500 ng/mL SY-treated group was further reduced to 34.5% and 30.2%, by combining with FO at concentration of 200 and 260 μM, respectively. These two concentrations of FO alone only slightly reduced the proliferation to 83.8% and 72.3%, respectively. This phenomenon was further confirmed by microscopic images of phase contrast and trypan blue staining. The shapes of 500 ng/mL SY-treated A549 cells almost became shrunken (Figure 1B), but most of them still excluded the trypan blue staining (Figure 1C), indicating the cell viability was only slightly affected. When combined with 200 μM FO, the cells were almost positively stained with trypan blue (Figure 1C), indicating the killing effect of SY was synergistically enhanced.

The synergism of this combination in suppressing the growth of A549 cells was determined by the combination index (CI) derived by Chou and Talalay, which quantitatively depict synergism (CI < 1), additive effect (CI = 1), and antagonism (CI > 1) [27]. As shown in Table 1, the CI values are all below 1, indicating synergisms of these two nutraceuticals in their antiproliferative effects. In contrast, the human fetal lung fibroblast MRC-5 cells were unsusceptible to this combination effect (Figure 1D), implying the selectivity of this synergism in lung adenocarcinoma (LADC) cells.

**Figure 2:**
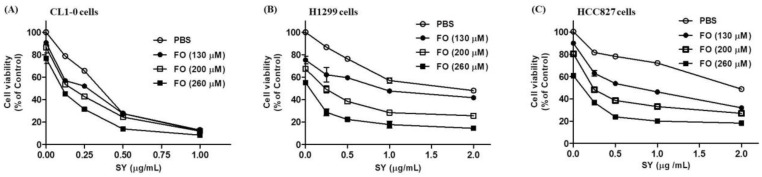
Combination effects of selenium yeast (SY) and fish oil (FO) on the cell viabilities of human lung adenocarcinoma CL1-0, H1299 and HCC827 cells. Cells were treated with the indicated concentrations of SY and FO for 72 h as described in materials and methods (Section 2). Cell viabilities of CL1-0 (**A**), H1299 (**B**) and HCC827 (**C**) cells were determined by sulforhodamine B binding assay. Data are expressed as mean ± standard error.

The wild-type p53 and Epidermal Growth Factor Receptor (EGFR) containing A549 cells are relatively insensitive to EGFR tyrosine kinase inhibitor like gefitinib [29]. To assess whether this synergism was a general phenomenon occurring in LADC cells, another three LADC cell lines, such as p53-null H1299, p53-mutant CL1-0 and gefitinib-sensitive HCC827 cells were tested. In these cell lines, the CL1-0 cells were relatively sensitive to SY. After 72 h of treatment, its proliferation could be suppressed to 27.6% of control by SY at concentration of 500 ng/mL (Figure 2A). Consistently, the less potent effects of lower concentrations SY in CL1-0 cells were dose-dependently enhanced by combining with FO (Figure 2A). Like in A549 cells, 500 ng/mL of SY only suppressed the viabilities of H1299 and HCC827 cells to 76.3% and 78.2% of control, respectively (Figure 2B,C). As expected, combination with FO dose-dependently enhanced the inhibitory effect of SY in H1299 (Figure 2B) and HCC827 (Figure 2C) cells. Further examining the synergisms by combination index (CI), most of the CI values in these three cell lines were all below 1 (Table 2, Table 3 and Table 4), except some potent concentrations of SY in CL1-0 and H1299 cells. These results suggest that the synergism is a general phenomenon in LADC cells.

### 3.2. SY and FO Act Synergistically to Induce Apoptosis of LADC Cells

**Figure 3:**
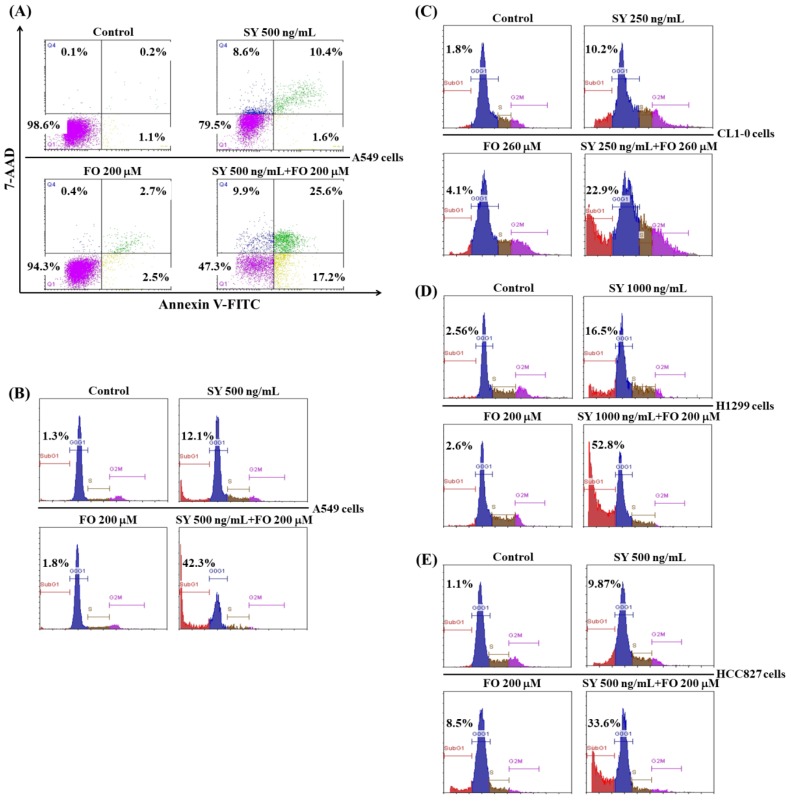
Combination effects of SY and FO on the apoptosis induction of human lung adenocarcinoma cells. Cells were treated with the indicated concentrations of SY and FO for 72 h as described in materials and methods (Section 2). The early and late apoptotic A549 cells analyzed by annexin V and 7-AAD staining and flow cytometry (**A**). The apoptotic sub-G1 fraction of A549 (**B**), CL1-0 (**C**), H1299 (**D**) and HCC827 (**E**) cells analyzed by propidium iodide staining and flow cytometry.

As previously mentioned, apoptosis induction is a critical event for both the anticancer activities of SY and FO. We next examined the apoptosis induction in cancer cells after treatment with SY and FO for 72 h. As described in the Materials and Methods (Section 2), the early apoptotic cells could be stained only by annexin V, whereas the late apoptotic cells could be stained by both annexin V and 7-amino-actinomycin D (7-AAD). Consistent with the result shown in Figure 1, Annexin V/7-AAD double staining assay demonstrated that both the percentages of early (17.2%, lower right quadrant) and late (25.6%, upper right quadrant) apoptosis of A549 cells induced by SY (500 ng/mL) combined with FO (200 μM) were much higher than the sums of respective individual treatment (Figure 3A). This synergistic apoptosis induction was further confirmed by propidium iodide staining to measure the percentage of cells with hypodiploid DNA contents (sub-G1 fraction) in flow cytometer. Consistently, combination of SY (500 ng/mL) and FO (200 μM) elevated the sub-G1 fraction from 1.3% in control group up to 42.3%, whereas the individual treatment only marginally increased to 12.1% and 1.8%, respectively (Figure 3B). Similar results were also observed in CL1-0, H1299 and HCC827 cells subjected to this combination at various concentrations. The sub-G1 percentages in combined-treatment groups were all markedly higher than the sums of the respective individual-treated groups (Figure 3C–E). This result was consistent with that shown in Figure 1 and Figure 2 and further displayed that FO synergized SY-induced apoptosis in LADC cell lines with different status of p53 and sensitivity to EGFR tyrosine kinase inhibitor.

### 3.3. Synergistic Activation of AMPK by SY and FO in A549 LADC Cells

**Figure 4:**
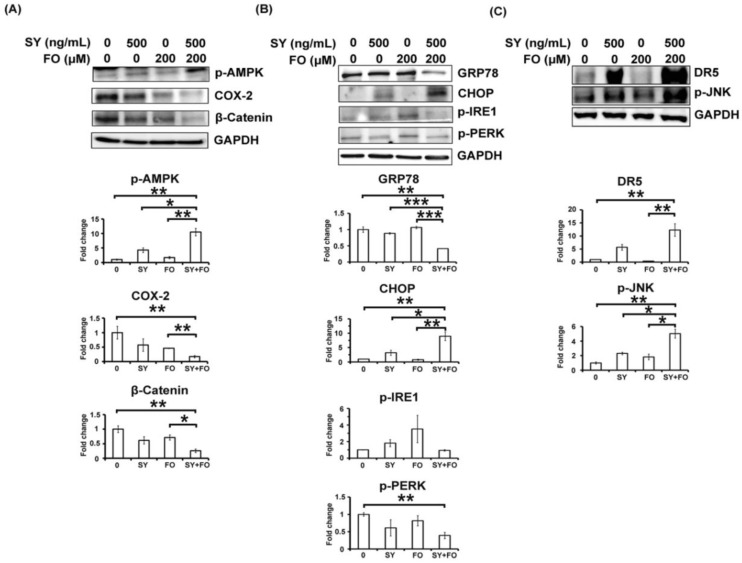
Combination effects of SY and FO on the activation of AMP-activated protein kinase (AMPK) and modulation of ER stress markers in human lung adenocarcinoma A549 cells. Cells were treated with the indicated concentrations of SY and FO for 72 h. Cell lysates were analyzed by Western blot, using Glyceraldehyde-3-Phosphate Dehydrogenase (GAPDH) as loading control. (**A**) Protein levels of phospho-AMPK (p-AMPK) and its downstream inhibitory targets, COX-2 and β-Catenin. (**B**) Protein levels of endoplasmic reticulum (ER) stress markers, CCAAT/enhancer-binding protein homologous protein (CHOP), glucose regulated protein of molecular weight 78 (GRP78), phospho-inositol-requiring enzyme 1 (p-IRE1) and phospho-protein kinase-like endoplasmic reticulum kinase (p-PERK). (**C**) Protein levels of the downstream effectors of CHOP-induced apoptosis death receptor-5 (DR5) and phospho-Jun amino-terminal kinase (p-JNK). Data presented are the representative Western blotting images and average fold changes in protein levels of three independent experiments. Values represent the means ± standard error of the mean (SEM) (*n* = 3). * *p* < 0.05; ** *p* < 0.01; *** *p* < 0.001.

As mentioned in introduction, AMPK had been shown to play the central role in the apoptosis inducing effects of both SY and FO. We examined the activation (phosphorylation) of AMPK (p-AMPK) in the treated A549 cells to investigate its involvement in this synergism. After 72 h of treatment, SY (500 ng/mL) or FO (200 μM) alone only exerted mild or non-significant effect, respectively, on the protein level of p-AMPK (Figure 4A). As expected, when treated with the combination of these two nutraceuticals, the p-AMPK protein was drastically elevated (Figure 4A). Consequently, both the downstream targets, such as β-catenin [10] and COX-2 [11], which had been reported to inhibit by selenium-activated AMPK were also suppressed in an according manner (Figure 4A). SY and FO appeared to enhance each other to trigger the AMPK-mediated apoptosis cascade.

### 3.4. Combination of SY and FO Elevates CHOP and Reduces GRP78 in A549 LADC Cells

As ER stress was involved in the apoptosis induction by either selenium [13,15] or the omega-3 fatty acid (DHA and EPA) contained in FO [23,24], we examined the changes of ER stress markers, such as CHOP, GRP78, phospho-inositol-requiring enzyme 1 (p-IRE1) and phospho-protein kinase-like endoplasmic reticulum kinase (p-PERK) during the synergistic apoptosis induction. Compared to the considerable amount of constitutively expressed pro-survival GRP78, the basal level of the pro-apoptotic CHOP in A549 cells was extremely low (Figure 4B). After 72 h of treatment, the increase of CHOP by SY (500 ng/mL) was observed, but its effect on the GRP78 was not significant, while FO (200 μM) did not significantly change the levels of CHOP and GRP78 (Figure 4B). These individual effects on the pro-apoptotic CHOP and pro-survival GRP78 proteins were consistent with the apoptosis induction by either alone, shown in Figure 3. Intriguingly, when these two nutraceuticals were combined, not only the pro-apoptotic CHOP was further drastically elevated, but also the unchanged pro-survival GRP78 was markedly reduced (Figure 4B). In contrast, the combination effect on other ER stress markers, such as p-IRE1 and p-PERK was relatively mild (Figure 4B). Among the components of ER stress/UPR response cascade, combination of SY and FO appeared to predominantly increase the ratio of CHOP to GRP78, leading to the synergistic apoptosis induction. In agreement, the downstream effectors of CHOP-induced apoptosis, such as death receptor-5 (DR5) and phospho-Jun amino-terminal kinase (p-JNK) [30] were also synergistically induced by the combination treatment (Figure 4C).

**Figure 5:**
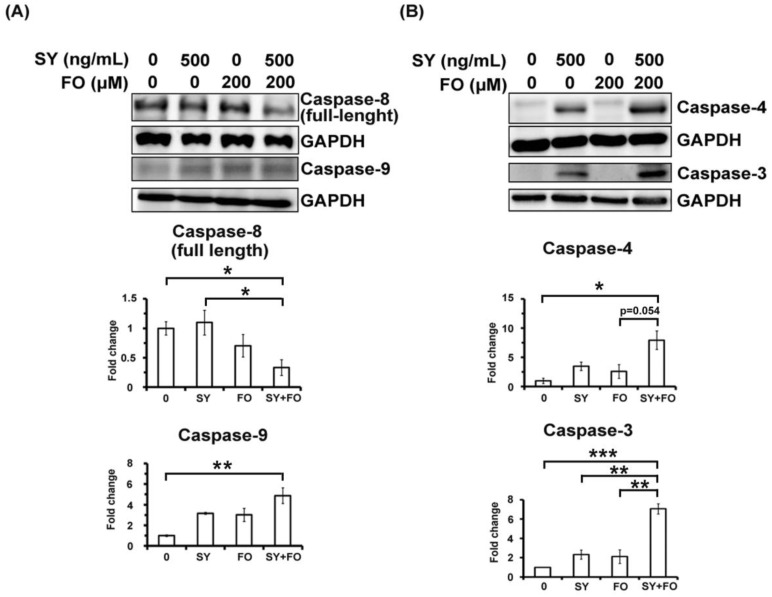
Combination effects of SY and FO on the activation of caspases in human lung adenocarcinoma A549 cells. Cells were treated with the indicated concentrations of SY and FO for 72 h. Cell lysates were analyzed by Western blot, using GAPDH as loading control. (**A**) Protein levels of full-length caspase-8 (extrinsic pathway) and cleaved caspase-9 (intrinsic pathway). (**B**) Protein levels of cleaved caspase-4 (ER stress-related) and caspase-3 (terminal executioner). Data presented are the representative Western blotting images and average fold changes in protein levels of three independent experiments. Values represent the means ± SEM (*n* = 3). * *p* < 0.05; ** *p* < 0.01; *** *p* < 0.001.

We next examined the activation of caspases, the executioners [31] of apoptosis. Upon persistent ER stress, CHOP had been shown to upregulate DR5 to induce the cleavage/activation of extrinsic apoptosis initiating caspase-8 [32]. In line with the induction of CHOP and DR5, the full-length caspase-8 was decreased in the combination-treated group (Figure 5A), indicating the activation of caspase-8. Besides, the intrinsic apoptosis initiating caspase-9 was mildly activated in treated groups (Figure 5A). According to expectation, the ER stress-related caspase-4 and the terminal executioner caspase-3 were both synergistically activated by combination of SY and FO (Figure 5B). The combination effects on the activation of caspase-4 further demonstrated the critical role of ER stress response in the synergistic apoptosis induction by these two nutraceuticals.

### 3.5. AMPK Inhibition Diminishes the Synergistic Apoptosis Induction and Caspase-4 Activation

**Figure 6:**
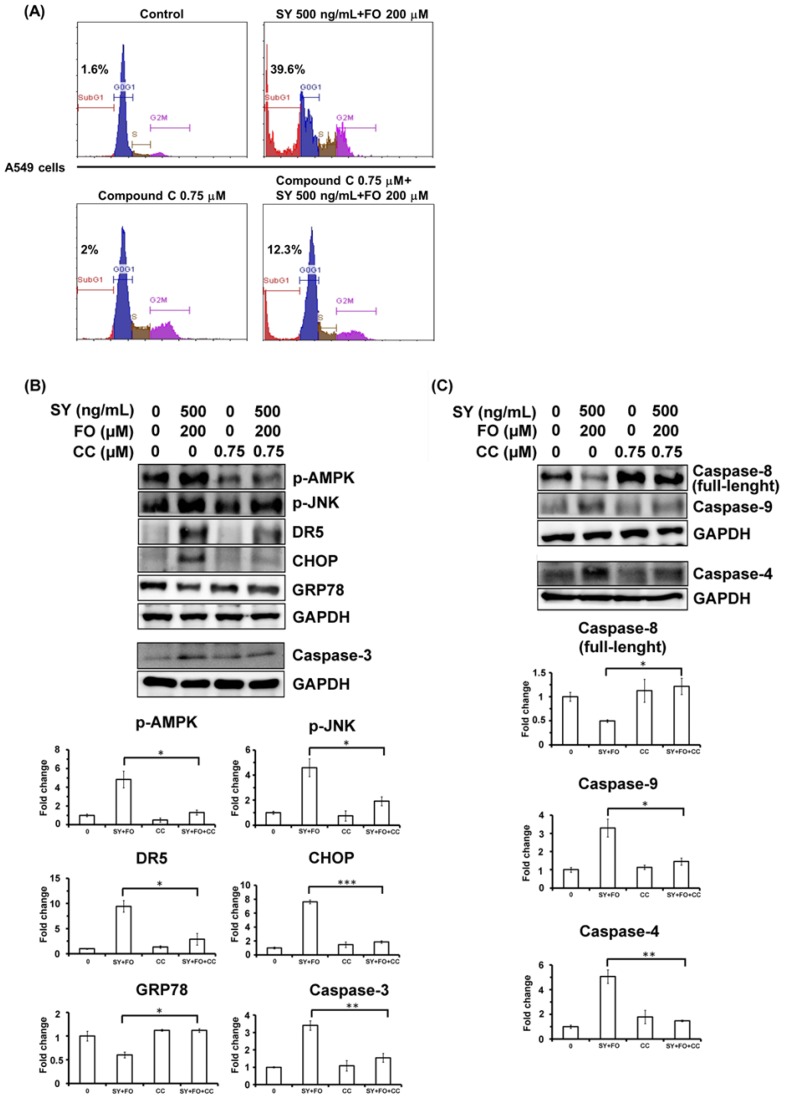
Effects of AMPK inhibitor compound C (CC) on the apoptosis induction and AMPK-ER stress cascade induced by SY combined with FO in A549 cells. Compound C was added to cells 1 h before the treatment with combination of SY and FO. (**A**) The apoptotic sub-G1 fraction was analyzed by propidium iodide staining and flow cytometry after treatment as indicated for 72 h. (**B**) After treatment as indicated for 48 h, the protein levels of phospho-AMPKα (p-AMPKα), phospho-JNK (p-JNK), DR5, CHOP, GRP78 and cleaved caspase-3 (terminal executioner) were analyzed by Western blot, using GAPDH as loading control. (**C**) After treatment as indicated for 72 h, the protein levels full-length caspase-8 (extrinsic pathway), cleaved caspase-9 (intrinsic pathway) and cleaved caspase-4 (ER stress-related) were analyzed by Western blot, using GAPDH as loading control. In (**B**,**C**), data presented are the representative Western blotting images and average fold changes in protein levels of three independent experiments. Values represent the means ± SEM (*n* = 3). * *p* < 0.05; ** *p* < 0.01; *** *p* < 0.001.

To further assess whether this synergistic apoptosis induction was AMPK-dependent, a selective AMPK inhibitor compound C (also called dorsomorphin) was added to the A549 cells 1 h before treatment with the combination of SY and FO. As shown in Figure 6A, the 39.6% apoptotic sub-G1 fraction induced by treatment with the combination for 72 h was reduced to 12.3% by 0.75 μM of compound C. After 48 h of treatment, it was observed that compound C (0.75 μM) antagonized the combination treatment-induced increase of p-AMPK, DR5, p-JNK and cleaved caspase-3 protein levels (Figure 6B). The elevated CHOP to GRP78 ratio was also reversed by the compound C (Figure 6B). In agreement, the activation of caspase-4, -8, -9 by combination of SY and FO was diminished by compound C at dose of 0.75 μM (Figure 6C). Combination of SY and FO appears to synergistically induce ER stress-mediated apoptosis of lung adenocarcinoma cells through AMPK activation.

## 4. Discussion

The effects of selenium in the prevention and integrative treatment of lung cancer have been widely investigated [3,4,5,6]. However, its clinical efficacy seems only superficially plausible, because some outcomes of clinical trials are unfavorable or even conflict [4,5]. One possible cause might be that the selenium compounds at clinically achievable concentration were not potent enough to essentially suppress the growth of lung cancer cells in most of the cases. The sensitivities of various lung cancer cells to the anticancer activities of selenium compounds are quite different in nature. In line with this, our results show that although SY inhibited the proliferation of all the four tested LADC cell lines, only the growth of CL1-0 cells could be vigorously suppressed at the concentration of 500 ng/mL, the upper limit of the mean plasma selenium levels associated with toxicity in human [28]. Even at concentration of 1000 ng/mL, the proposed upper tolerated limit [28], the majority of the tested lung cancer cells still survived and kept proliferating. To achieve greater clinical efficacy, identifying an effective combination is emerging as an attractive strategy. As apoptosis induction through ER stress cascade is crucial for the anticancer activity of selenium [8,15], dietary nutraceutical with complementary mechanism of action in modulating the cascade is particularly engaging for this purpose.

Upon ER stress induced by selenium in cancer cells, the pro-survival ER chaperon GRP78 will be induced to rescue the cells from pro-apoptotic response followed by CHOP expression [13,15]. It has been shown that in selenium-treated cancer cells, overexpression of GRP78 diminished selenium-induced CHOP and the subsequent apoptosis [15], whereas knockdown of GRP78 further raised the CHOP expression and enhanced the effect of selenium [33]. In support of this, our result shows that the SY-induced CHOP expression was further enhanced by combination with FO, while the GRP78 was markedly reduced. It had been shown in cancer cells that DHA decreased GRP78 and activated caspase-4 [24], and EPA increased CHOP [34], respectively. Considering this complimentary mechanism in modulation of ER stress cascade, it is rational to choose the DHA and EPA enriched FO for synergizing the SY-induced apoptosis of cancer cells. Although FO alone did not significantly change the GRP78 and CHOP protein levels at the concentration used in this study, the two proteins were oppositely regulated when combined with SY, resulting in augmentation of ER stress-mediated apoptosis in LADC cells.

Consistent with that shown by Wu et al. in prostate cancer cells [15], we found the basal level of CHOP in A549 cells was much lower than the considerable amount of constitutively expressed GRP78. It had been reported that many tumor cell lines and primary clinical samples express permanently elevated levels of GRP78 [35], which might promote metastasis [36], inhibit caspase-4 [24] and pro-apoptotic pathways, support chemoresistance and thereby worsen the prognosis [35]. Regarding the reduction of GRP78 by combination of SY and FO in A549 cells, further studies are warranted to explore the potentiality of this combination in alleviating the chemoresistance and metastasis, as well as improving the prognosis of LADC patients.

In accordance with the central role of AMPK reported in the anticancer activities of selenium [10,11,12] and FO ingredients (DHA and EPA) [16,18,21,22], we found the AMPK of combination-treated cells was synergistically activated in the same manner as observed either in the elevation of CHOP to GRP78 ratio or the activation of caspase-4 and -3. The diminishment of both synergistic apoptosis induction and elevated CHOP/GRP78 ratio by compound C implied the actual involvement of AMPK in the pro-apoptotic regulation of CHOP and GRP78 in LADC cells. In parallel, similar opposite regulation of CHOP and GRP78 by AMPK activation was also observed by Leclerc et al. in metformin-treated acute lymphoblastic leukemia lymphoblasts [37]. Mechanistically, their report provides further evidence to support the AMPK-mediated ER stress-apoptosis we observed. However, the effective concentration of metformin used in the report of Leclerc et al. was as high as 2.5 mM [37], which is far in excess of the maximum plasma concentration for metformin in healthy subjects (8–16 μM) [38]. To achieve this peculiar effect of AMPK, the effective concentrations of SY and FO ingredients (DHA and EPA) for the synergism shown in this study were both clinically achievable [28,39]. Compared to the effect exerted by 2.5 mM metformin, the synergistic combination effect of SY and FO might be more clinically applicable.

Previous studies had shown the inhibition of COX-2 [11] and β-catenin [10] by selenium-activated AMPK in cancer cells. The synergistically activated AMPK by SY and FO was also accompanied with an according inhibition of these two oncoproteins. Whether this inhibition of COX-2 and β-catenin was attributed to the AMPK-mediated ER stress modulation is remained to be elucidated. Of noted, COX-2 and β-catenin were proposed to be the targets for selenium to suppress the epithelial-mesenchymal transition (EMT) and stemness of cancer cells [40]. The synergism we found might also effective on the inhibition of EMT and cancer stem traits. Further study has been undertaken to evaluate this deduction.

Besides lung cancers, selenium has also been widely proposed for the prevention and integrative treatment of many cancers [3,6]. Previous studies had shown the enhanced selenium-anticancer activities by combining with vitamin E and green tea in prostate and colon cancer cells, respectively [14,41]. In the study of vitamin E and selenium combination, the disruption of ER microenvironment in selenium-treated cancer cells was found [14]. Moreover, the activation of AMPK by vitamin E and green tea had been respectively reported [42,43] and the contribution of ER stress and caspae-4 to vitamin E-induced apoptosis of cancer cells had also been demonstrated [44]. As such, the AMPK-ER stress cascade demonstrated in the present study might also participate in the enhanced selenium-anticancer activities by vitamin E or green tea mentioned above. Furthermore, the synergism between SY and FO might be effective in prostate, colon and other cancer cells. Both inferences are worthy of further investigation for the effective use of these two popular nutraceuticals.

In addition to the inhibitory effects in cancer cells, combination of SY and FO had also been reported by Wang et al. to synergistically reduce the splenic immunosuppressive cells and enhance the anti-tumor immunity [45]. In that report, a higher apoptotic caspase-3 was shown in the tumor treated with combination of SY and FO as compared with that treated with either alone. Although this phenomenon was not noted or mentioned by Wang et al. in their report [45], it is in accordance with our findings shown in the present study. Hence, the synergistic apoptotic induction by SY and FO might occur in that immunocompetent lung carcinoma mouse model used by Wang et al. [45].

At the physiological concentrations, either SY or FO alone might not be potent enough to substantially exert their expected anticancer activities in the most clinical cases. When combined together, these two nutraceuticals could enhance each other to activate AMPK and induce ER stress-mediated apoptosis of cancer cells. Through the synergistic inhibition of COX-2 and β-catenin, this combination might also suppress the EMT and cancer stem traits, which closely related to the tumor therapeutic resistance [46]. Along with the previously reported enhancement in anti-tumor immunity [45], the synergistic combination of SY and FO might be effective not only in the prevention, but also in the integrative treatment of cancers. The insusceptibility of human fetal lung fibroblast MRC-5 cells to this synergism implies the selectivity of this combination effect. After satisfactory preclinical assessment to ensure the safety and potency, it would be of great interest to conduct clinical trials to evaluate the combination use of SY and FO in the prevention and integrative treatment of LADC.

## 5. Conclusions

As illustrated in the Graphical Abstract, our results demonstrate that FO synergizes SY-induced apoptosis of human lung adenocarcinoma cells through activation of AMPK and the subsequent opposite regulation of GRP78 and CHOP. With this synergistic combination effect, the optimized regimen of SY combined with FO may have potential in the prevention and integrative treatment of LADC.

## Figures and Tables

**Table 1:** Combination index (CI) values of selenium yeast (SY) and fish oil (FO) combinations vs. the inhibition (FA, fraction affected) of A549 cell viabilities. Each gram of FO contains 220 mg docosahexaenoic acid (DHA) and 330 mg eicosapentaenoic acid (EPA). The concentration of FO represents its content of omega-3 fatty acid (DHA + EPA). Values below 1 indicate synergistic effects, whereas those equal or close to 1 are additive and those above 1 are antagonistic.

SY (ng/mL)	FO (μM)	FA	CI
250	130	0.29	0.945
250	200	0.36	0.968
250	260	0.44	0.983
500	130	0.43	0.840
500	200	0.65	0.576
500	260	0.70	0.631
1000	130	0.51	0.969
1000	200	0.69	0.650
1000	260	0.72	0.711
2000	130	0.63	0.999
2000	200	0.72	0.813
2000	260	0.80	0.683

**Table 2:** CI values of SY and FO combinations vs. the inhibition of CL1-0 cell viabilities. Each gram of FO contains 220 mg DHA and 330 mg EPA. The concentration of FO represents its content of omega-3 fatty acid (DHA + EPA). Values below 1 indicate synergistic effects, whereas those equal or close to 1 are additive and those above 1 are antagonistic.

SY (ng/mL)	FO (μM)	FA	CI
125	130	0.43	0.740
125	200	0.46	0.565
125	260	0.55	0.738
250	130	0.52	0.974
250	200	0.57	0.950
250	260	0.68	0.775
500	130	0.73	0.990
500	200	0.76	0.950
500	260	0.86	0.664
1000	130	0.88	1.009
1000	200	0.88	1.040
1000	260	0.92	0.810

**Table 3:** CI values of SY and FO combinations vs. the inhibition of H1299 cell viabilities. Each gram of FO contains 220 mg DHA and 330 mg EPA. The concentration of FO represents its content of omega-3 fatty acid (DHA + EPA). Values below 1 indicate synergistic effects, whereas those equal or close to 1 are additive and those above 1 are antagonistic.

SY (ng/mL)	FO (μM)	FA	CI
250	130	0.38	0.856
250	200	0.51	0.347
250	260	0.71	0.468
500	130	0.62	0.607
500	200	0.78	0.389
500	260	0.41	0.993
1000	130	0.52	0.955
1000	200	0.72	0.529
1000	260	0.82	0.381
2000	130	0.58	1.208
2000	200	0.74	0.697
2000	260	0.85	0.422

**Table 4:** CI values of SY and FO combinations vs. the inhibition of HCC827 cell viabilities. Each gram of FO contains 220 mg DHA and 330 mg EPA. The concentration of FO represents its content of omega-3 fatty acid (DHA + EPA). Values below 1 indicate synergistic effects, whereas those equal or close to 1 are additive and those above 1 are antagonistic.

SY (ng/mL)	FO (μM)	FA	CI
250	130	0.37	0.706
250	200	0.52	0.675
250	260	0.63	0.700
500	130	0.46	0.677
500	200	0.61	0.612
500	260	0.76	0.549
1000	130	0.54	0.694
1000	200	0.67	0.603
1000	260	0.8	0.521
2000	130	0.68	0.575
2000	200	0.73	0.604
2000	260	0.82	0.535

## Abbreviations

AMPK	AMP-activated protein kinase
CC	compound C
CHOP	CCAAT/enhancer-binding protein homologous protein
DR5	death receptor-5
EGFR	epidermal growth factor receptor
ER	endoplasmic reticulum
FO	fish oil
GRP78	glucose-regulated protein 78
p-IRE1	phospho-inositol-requiring enzyme 1
LADC	Lung adenocarcinoma
SEM	standard error of the mean
SY	selenium yeast
p-JNK	phospho-Jun amino-terminal kinase
p-PERK	phospho-protein kinase-like endoplasmic reticulum kinase
UPR	unfolded protein response
